# Lipidomic Analysis Links Mycobactin Synthase K to Iron Uptake and Virulence in *M*. *tuberculosis*

**DOI:** 10.1371/journal.ppat.1004792

**Published:** 2015-03-27

**Authors:** Cressida A. Madigan, Amanda Jezek Martinot, Jun-Rong Wei, Ashoka Madduri, Tan-Yun Cheng, David C. Young, Emilie Layre, Jeffrey P. Murry, Eric J. Rubin, D. Branch Moody

**Affiliations:** 1 Division of Rheumatology, Immunology and Allergy, Brigham and Women’s Hospital, Harvard Medical School, Boston, Massachusetts, United States of America; 2 Department of Immunology and Infectious Diseases, Harvard School of Public Health, Boston, Massachusetts, United States of America; National Institutes of Health, UNITED STATES

## Abstract

The prolonged survival of *Mycobacterium tuberculosis* (M. tb) in the host fundamentally depends on scavenging essential nutrients from host sources. M. tb scavenges non-heme iron using mycobactin and carboxymycobactin siderophores, synthesized by mycobactin synthases (Mbt). Although a general mechanism for mycobactin biosynthesis has been proposed, the biological functions of individual *mbt* genes remain largely untested. Through targeted gene deletion and global lipidomic profiling of intact bacteria, we identify the essential biochemical functions of two mycobactin synthases, MbtK and MbtN, in siderophore biosynthesis and their effects on bacterial growth *in vitro* and *in vivo*. The deletion mutant, Δ*mbtN*, produces only saturated mycobactin and carboxymycobactin, demonstrating an essential function of MbtN as the mycobactin dehydrogenase, which affects antigenicity but not iron uptake or M. tb growth. In contrast, deletion of *mbtK* ablated all known forms of mycobactin and its deoxy precursors, defining MbtK as the essential acyl transferase. The *mbtK* mutant showed markedly reduced iron scavenging and growth *in vitro*. Further, Δ*mbtK* was attenuated for growth in mice, demonstrating a non-redundant role of hydroxamate siderophores in virulence, even when other M. tb iron scavenging mechanisms are operative. The unbiased lipidomic approach also revealed unexpected consequences of perturbing mycobactin biosynthesis, including extreme depletion of mycobacterial phospholipids. Thus, lipidomic profiling highlights connections among iron acquisition, phospholipid homeostasis, and virulence, and identifies MbtK as a lynchpin at the crossroads of these phenotypes.

## Author Summary

*M*. *tuberculosis* (M. tb) survives only if it can acquire iron from its human host, so new therapies for tuberculosis might be discovered if the pathways necessary for iron acquisition are identified. M. tb scavenges iron in two ways: from free iron, or from the blood in the form of heme. To bind free iron, M. tb uses mycobactin, a lipopeptide that tightly binds iron and transports it to the bacterial cytosol. Mycobactin is thought to be required for M. tb virulence, but its biosynthesis is incompletely understood. To investigate mycobactin biosynthesis, we deleted the *mbtN* and *mbtK* genes potentially required for generating the mycobactin lipid tail. Then, an organism-wide screen of lipids identified changed molecules that are the direct targets of these genes or have broader downstream functions. MbtK deletion specifically changed the lipid component of mycobactin and created extreme iron-deprivation that prevented growth of M. tb in mice. Unexpectedly, the combination of MbtK loss and iron starvation triggered a global depletion of phospholipids, a key constituent of the bacterial membrane. These studies establish that mycobactins, acting independently of the heme acquisition pathway, impact lipid homeostasis and M. tb survival, supporting efforts to develop host-directed therapies for tuberculosis.

## Introduction

*Mycobacterium tuberculosis* (M. tb), an obligate human pathogen, causes tuberculosis, typically through a decades-long infection. Long-term survival in the host necessitated the evolution of mechanisms for scavenging essential nutrients. Iron plays crucial roles in bacterial respiration and DNA synthesis, and its availability affects the outcome of natural tuberculosis infection in humans. Thus, targeting of iron status has been considered as a host-directed approach to treating tuberculosis [1–3], which increases the need to understand non-redundant pathways of iron acquisition by this pathogen. M. tb acquires heme-bound or non-heme iron from the host using two separate pathways. A recently identified hemophore uptake system imports iron bound to its native host substrate, heme [4,5]. Non-heme iron present in host stores is typically bound to proteins, such as transferrin and ferritin. These proteins chelate free iron by binding it with high affinity, thereby sequestering iron from infectious organisms [6]. To acquire non-heme iron, M. tb synthesizes two structurally related, hydroxamate polyketide-polypeptide siderophores: mycobactin and carboxymycobactin (also known as exochelin) [7,8].

The importance of mycobactin and carboxymycobactin to M. tb virulence has been suggested by impaired growth of a mycobactin mutant in cultured macrophages [9,10] and impaired growth *in vitro* and *in vivo* of M. tb mutants in the siderophore export apparatus [10–12]. Further, mycobactin biosynthesis genes are up-regulated during infection of macrophages, and three genes (*mbtB*, *mbtE*, and *mbtG*) are required for *in vitro* growth [10,13–15]. The necessity of iron scavenging is unquestioned; however, the relative importance during infection of iron acquisition by mycobactin versus the hemophore system remains unknown.

Mycobactin is biosynthesized by mycobactin synthases (Mbt), a family of non-ribosomal peptide synth(et)ases. Their proposed functions in mycobactin synthesis were assigned based on sequence homology to enzymes of known function, and transcriptional repression in iron-replete conditions by the iron-responsive repressor, IdeR [16–18]. Bioinformatics approaches predict a theoretical biosynthetic mechanism whereby the polyketide-polypeptide backbone is acylated. Of the 14 proposed mycobactin synthases, only MbtB, MbtD, MbtE and MbtG have been directly tested for their non-redundant functions in live M. tb [4,9,10,15], while others have been evaluated *in vitro* with recombinant enzymes and artificial substrates or in other mycobacterial species [16,19–21]. These studies suggest that two mycobactin synthases, MbtK and MbtN, may function as a mycobactin acyl transferase and dehydrogenase, respectively. These two enzymes are thought to transfer an acyl chain to the ε-amino group of a lysine-containing polyketide-polypeptide, and introduce an unsaturation into fatty acids destined for mycobactin incorporation [16,19,22]. However, the non-redundant functions of MbtK and MbtN and their roles in mycobacterial growth *in vitro* or *in vivo* have not been investigated.

Mycobactin’s long, monocarboxyl tail increases its hydrophobicity, promoting adherence to M. tb’s lipidic surface, and likely plays a role in transport across lipid membranes. In contrast, the short, dicarboxyl tail of carboxymycobactin increases its water-solubility, allowing it to be released from the cell surface into aqueous biological solutions. Iron acquisition is thought to occur through initial interaction of iron with carboxymycobactin, followed by transfer of iron to form mycobactin-iron complexes, which convert cationic free iron into a neutral, lipid-linked complex that can pass through two mycobacterial membranes to the cytosol [2,17]. Therefore, we sought to determine the roles of lipid-modifying enzymes, MbtN and MbtK, in iron uptake. Hydroxamate siderophores carry a *cis* unsaturation at C2-3 in the fatty acyl unit [23,24]. The unsaturation with this particular location and stereochemistry is highly characteristic of mycobactins and absent from unsaturated fatty acids found in other acylated mycobacterial macromolecules. This unique modification renders unsaturated dideoxymycobactin, a mycobactin precursor, 40 times more antigenic for human T cells, as compared to saturated dideoxymycobactin [25]. Evolutionary conservation of this unsaturation suggests a possible biological role in iron scavenging, but this has not been tested experimentally.

We investigated the function of MbtN and MbtK in intact M. tb, where we could observe their biological roles without prior knowledge of their natural substrates or products. These studies rely on new methods of comparative lipidomics, whereby high performance liquid chromatography-mass spectrometry (HPLC-MS) detects many thousands of metabolites in one experiment, including both named molecules and unnamed molecules with a unique mass or *m/z* value [15,26–28]. Because the lipidomics platform simultaneously analyzes both expected lipid products and nearly all lipids in the cell, this unbiased approach can measure downstream effects on expected substrates and lipids with no known relationship to the enzyme. After generating mutants with single gene deletions at *mbtK* or *mbtN*, both enzymes were found to be essential for modification of siderophore lipids. However, only MbtK is essential for growth *in vitro* and *in vivo*. Using the broader detection enabled by the lipidomic approach, we discovered unexpected contributions of mycobactins and iron starvation to membrane phospholipid maintenance.

## Results

### Lipidomic profiling of mycobactin synthase mutants

**Fig 1:**
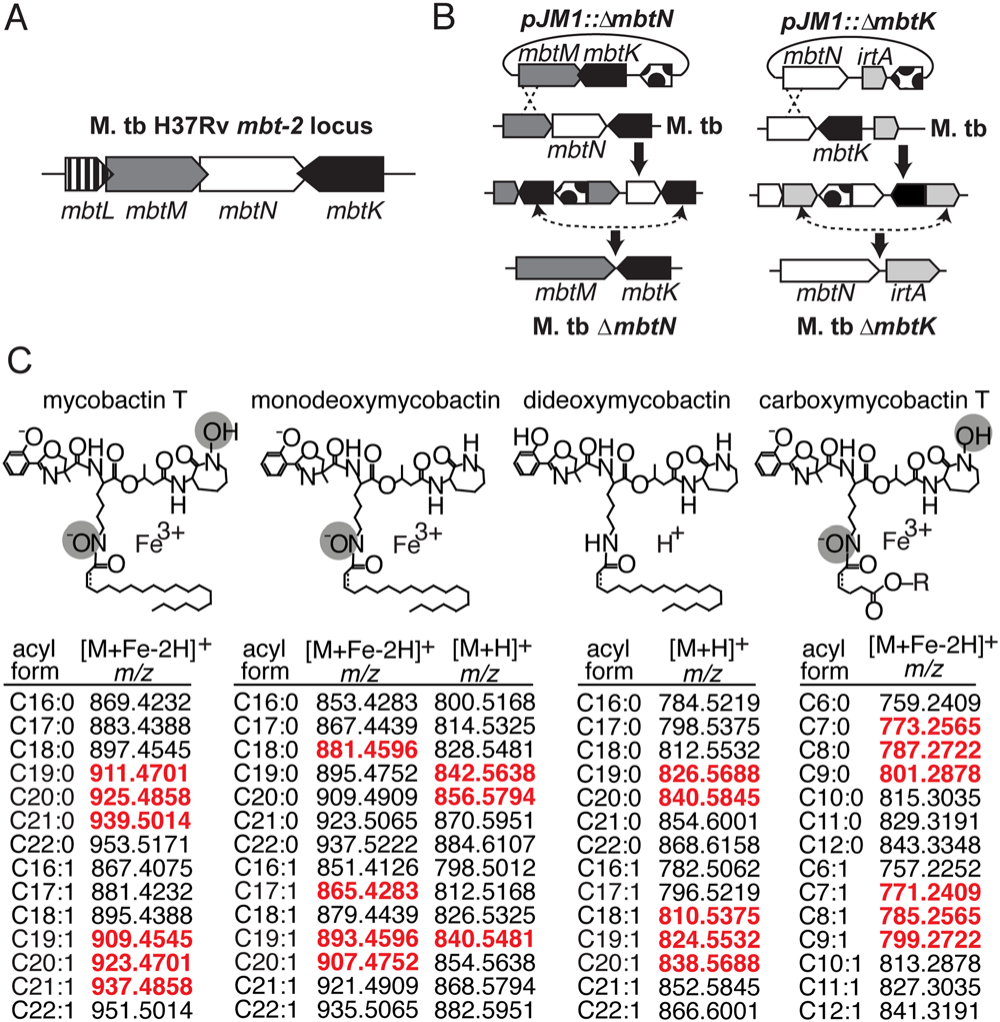
Mycobactin synthases synthesize M. tb siderophores mycobactin and carboxymycobactin. (**A**) The *mbt-2* locus encodes mycobactin synthases that generate and modify the lipidic portion of mycobactin and carboxymycobactin. (**B**) M. tb mutants in *mbtN* and *mbtK* were generated by homologous recombination between pJM1::Δ*mbtN* or pJM1::Δ*mbtK* and flanking regions. Dots, *hyg* hygromycin resistance cassette and *sacB Bacillus subtilis* levansucrase gene. (**C**) The structures mycobactin T, monodeoxymycobactin, dideoxymycobactin and carboxymycobactin T are shown with the potential unsaturation indicated by a dashed line. In addition to the depicted core structures, known variations in the fatty acyl chain, listed below as *m/z* values of [M+H]^+^ or [M+Fe-2H]^+^ adducts, were calculated and used to search the lipidomics datasets. Monodeoxymycobactin forms both [M+H]^+^ and [M+Fe-2H]^+^ adducts. Apo-mycobactin was not detected. For carboxymycobactin, R = H or CH_3_. Detected ions, highlighted in red, are highlighted in scatter plots in Fig. 2A and 3A.

The genes *mbtK* and *mbtN* are likely part of the natural iron scavenging response of M. tb, based on prior studies showing their transcriptional de-repression in iron-depleted medium [16]. Like other enzymes predicted to synthesize the lipid tails of mycobactin and carboxymycobactin, MbtK and MbtN are encoded by the M. tb *mbt-2* locus (Fig. 1A). *In vitro*, recombinant MbtK transfers fatty acids to lysine acceptors on synthetic mycobactin-like peptides, and MbtN reduces fatty acids, suggesting that these enzymes might catalyze similar reactions during mycobactin biosynthesis [16,21]. However, the necessity or sufficiency of these enzymes to mycobactin biosynthesis in intact M. tb is unknown. Further, the functions of MbtK and MbtN in biosynthesis of carboxymycobactin and deoxymycobactins, including the dideoxymycobactin antigen for human T cells [25], have not been investigated. To generate mutants in M. tb H37Rv, regions of approximately 1 kilobase flanking *mbtN* or *mbtK* were amplified, fused by polymerase chain reaction (PCR) and cloned into the suicide plasmid, pJM1 (Fig. 1B) [29,30]. Transformants were selected with hygromycin and counterselected with sucrose. Mycobactin J (2 μg/ml) was added *in trans* during cloning to ensure the initial survival and recovery of mycobactin-deficient mutants. After confirming mutations (S1 Fig), the loci were complemented with integrating plasmid pGH1000A expressing *mbtN* or *mbtK* constitutively under a mycobacterial *groEL* promoter [31].

**Fig 2:**
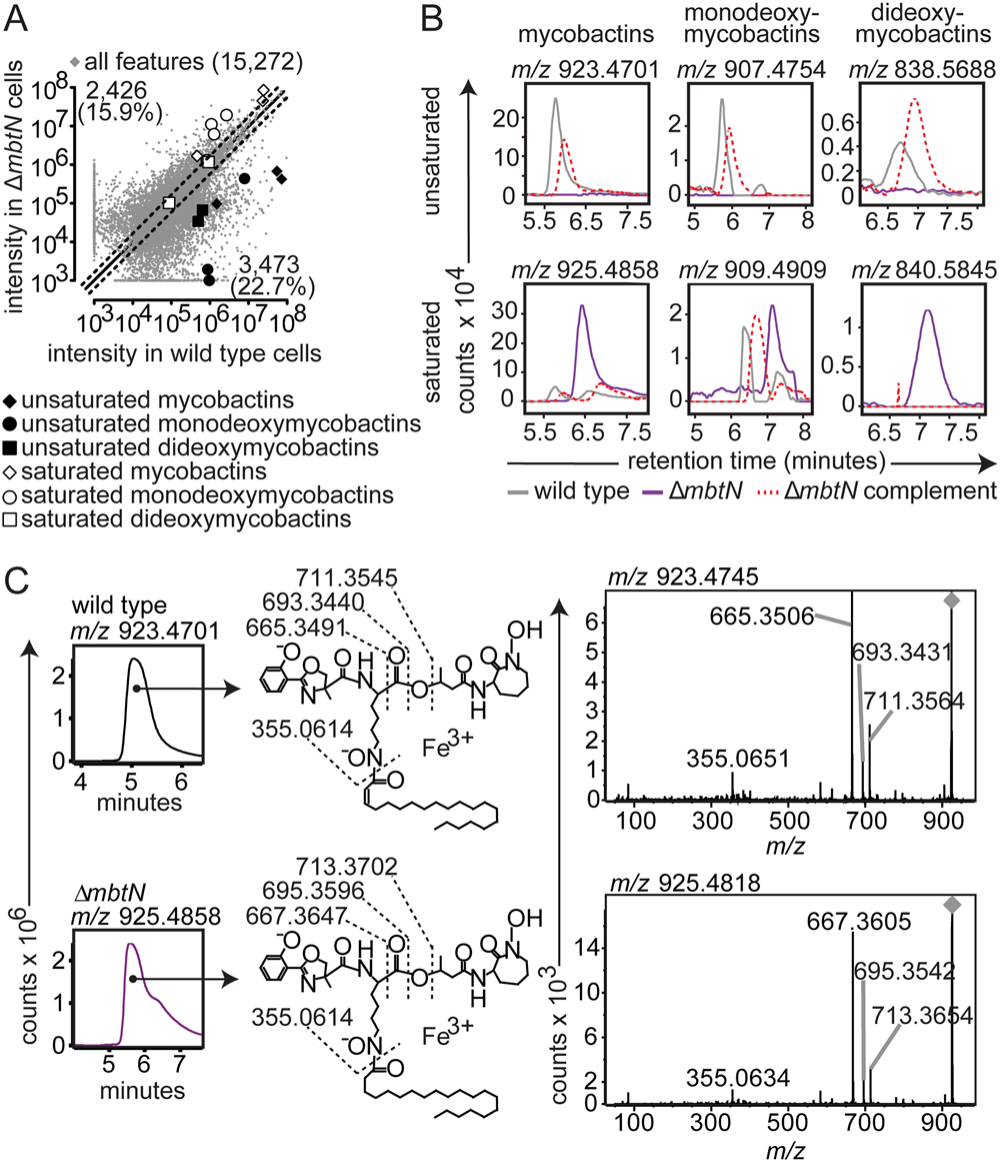
Δ*mbtN* lacks unsaturated mycobactin. (**A**) Acetone precipitation of lipids from three replicate cultures grown on iron-depleted agar were detected by three independent runs on reversed phase HPLC-MS to generate aligned datasets in XCMS software. Features increased or decreased by ≥ two-fold fall outside the dashed lines. Highlighted ions match the highlighted calculated *m/z* values in Fig. 1C, corresponding to: saturated mycobactins (white diamonds; *m/z* 911.4739, *m/z* 925.4883 and *m/z* 939.5008), unsaturated mycobactins (black diamonds; *m/z* 909.4564, *m/z* 923.4726 and *m/z* 937.4869), saturated monodeoxymycobactins (white circles; *m/z* 842.5663, *m/z* 856.5809, *m/z* 856.5828 and *m/z* 881.4590), unsaturated monodeoxymycobactins (black circles; *m/z* 865.4293, *m/z* 893.4444, and *m/z* 907.4781), saturated dideoxymycobactins (white squares; *m/z* 826.5531 and *m/z* 840.5852), and unsaturated dideoxymycobactins (black squares; *m/z m/z* 824.5594 and *m/z* 838.5714). All molecules detected as [M+Fe-2H]^+^, except *m/z* 856.5794 and dideoxymycobactins, detected as [M+H]^+^. (**B**) Representative ion chromatograms from three replicates from wild type (grey), Δ*mbtN* (purple) and Δ*mbtN* complement (dashed red) corresponding to unsaturated mycobactin *m/z* 923.4701, monodeoxymycobactin *m/z* 907.4754 and dideoxymycobactin *m/z* 838.5688; saturated mycobactin *m/z* 925.4858, monodeoxymycobactin *m/z* 909.4909 and dideoxymycobactin *m/z* 840.5845 (not detectable above background in wild type). (**C**) Collisional MS of unsaturated mycobactin (*m/z* 923.4701) from wildtype M. tb and saturated mycobactin (*m/z* 925.4858) from Δ*mbtN*. The constant presence of *m/z* 355, corresponding to the peptide-polyketide backbone, accompanied by a characteristic two-mass unit difference in unsaturated and saturated fragments separating the phenoloxazoline and acyl chain from the cobactin (*m/z* 711 and 713), isolate the mass difference to the fatty acyl unit.

**Fig 3:**
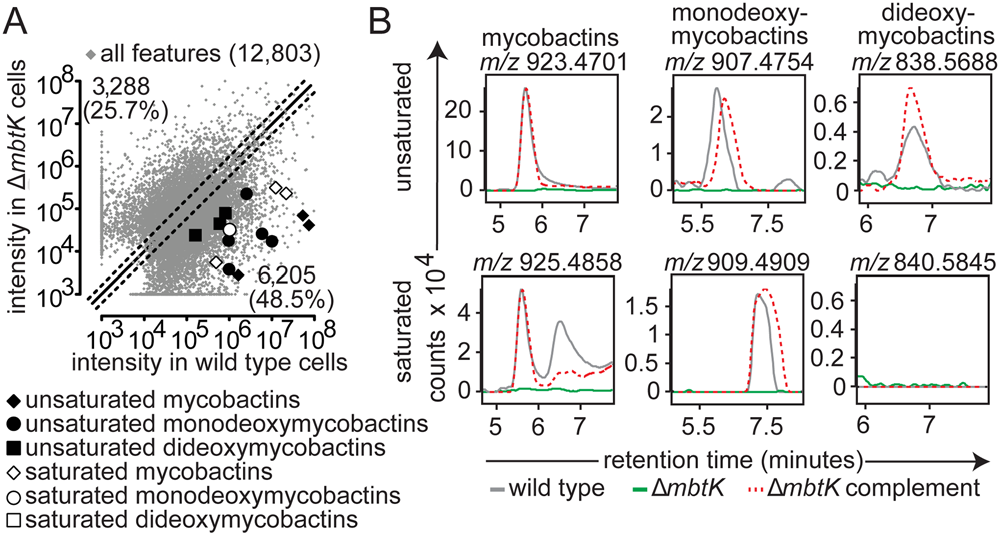
*mbtK* is required for mycobactin biosynthesis. (**A**) Three biological replicates of wild type M. tb or Δ*mbtK* were grown on iron-depleted agar and represented as in Fig. 2A. Ions highlighted correspond to saturated mycobactins (white diamonds; *m/z* 911.4753, *m/z* 925.4799 and *m/z* 939.4877), unsaturated mycobactins (black diamonds; *m/z* 909.4624, *m/z* 923.4782 and *m/z* 937.4869), saturated monodeoxymycobactin (white circle; *m/z* 856.5665), unsaturated monodeoxymycobactins (black circles; *m/z* 840.5532, *m/z* 865.4293, *m/z* 893.4444, *m/z* 893.4641 and *m/z* 907.4783), and unsaturated dideoxymycobactins (black squares; *m/z* 810.5370, *m/z* 824.5598 and *m/z* 838.5714). (**B**) Representative ion chromatograms from three replicates corresponding to unsaturated mycobactin *m/z* 923.4701, monodeoxymycobactin *m/z* 907.4754, and dideoxymycobactin *m/z* 838.5688; saturated mycobactin *m/z* 925.4858 monodeoxymycobactin *m/z* 909.4909, and dideoxymycobactin *m/z* 840.5845 (not detectable above background).

Mycobactin binds to the cell surface, but carboxymycobactin is released into aqueous media, so the two molecules were isolated from bacteria grown on solid or liquid media, respectively. To stimulate mycobactin production with iron starvation, we cultivated M. tb on iron-depleted agar medium [18]. Colonies grown on triplicate plates were removed by scraping, treated with chloroform:methanol to extract total lipids and then treated with cold (4°C) acetone to precipitate phospholipids and allow enrichment of mycobactin-like molecules in supernatants. Acetone soluble lipids were analyzed by reversed phase high performance liquid chromatography-mass spectrometry (HPLC-MS) in the positive mode. For carboxymycobactin, triplicate cultures were grown in iron-depleted liquid medium supplemented with ferric chloride to late log phase, washed and inoculated into unsupplemented iron-depleted medium to induce an iron-starved state [32]. After two weeks of conditioning, supernatants were filtered and extracted with ethyl acetate prior to reversed phase HPLC-MS analysis [15].

Analysis took advantage of a metabolomics platform in which ion monitoring over a large dynamic range of intensity allows nearly simultaneous tracking of compounds present at low and high concentrations within complex lipid mixtures. Chromatographic pre-separation prior to MS analysis allowed separate detection of compounds ranging in polarity from highly hydrophobic neutral lipids to highly polar glycolipids under conditions that minimize cross-suppression [28]. Injection of acetone-soluble lipids from one bacterial culture generated over 10,000 distinct ion intensity measurements. Each ion detected in at least two of three replicates corresponds to one “molecular event,” which is comprised of three linked values: accurate mass, retention time and intensity.

### Targeted and untargeted lipidomic analyses

We analyzed the expected precursors and products of MbtN and MbtK, as well as performed unbiased analysis of all 15,272 molecular events detected. For targeted analysis, we used the MycoMap to deduce the accurate masses corresponding to the molecular variants in four classes: mycobactins, monodeoxymycobactins, dideoxymycobactins, and carboxymycobactins [28]. Based on known patterns of molecular variation in acyl chain length and saturation, we deduced the accurate mass of 14 lipids within each class (Fig. 1C), then tracked the intensity of these seventy targets after gene deletion and iron depletion. Untargeted analysis is based on alignment of all events in the wild type and mutant datasets, a process whereby all events with matching masses and retention times are aligned using XCMS software [28,33]. Then, mean intensity ratios measured with or without genes and iron supplementation are measured, providing an organism-wide screen describing the percentage of all detected events that change by at least two-fold after deletion of *mbtN* (Fig. 2) or *mbtK* (Fig. 3). Prior analyses validate that the false positive rate for detecting compounds that are significantly changed within the cell wall is less than 1 percent [28].

### MbtN is the mycobactin/carboxymycobactin dehydrogenase

Because MbtN is predicted to have dehydrogenase function, a selective loss of unsaturated mycobactin-like metabolites would be expected in Δ*mbtN*. Therefore, we interrogated molecular events with the expected mass of mycobactins and deoxymycobactins carrying either saturated or unsaturated fatty acyl units in the parental strain and Δ*mbtN* (Fig. 2A). The assignment of unsaturated mycobactins is preliminarily made on masses that match the predicted *m/z* and retention time of intact unsaturated mycobactins (Fig. 1C). These assignments were confirmed through CID-MS analysis of products that show equivalent mass of the peptide unit (*m/z* 355.065) and multiple cleavage products corresponding to peptide fragments carrying either saturated (*m/z* 713. 370, 695.360, 667.365) or unsaturated (*m/z* 711.255, 693.343, 665.349) fatty acyl units (Fig. 2C). Ruling in this model, targeted analysis of Δ*mbtN* found these lipid classes were produced with masses matching saturated, but not unsaturated, fatty acids (Fig. 2A, white shapes). In contrast, events corresponding to unsaturated and saturated forms of mycobactin-like molecules were produced in the wild type parent (Fig. 2A, black shapes).

Whole organism-lipidomics is a relatively new method that uses computer-assisted methods to measure mycobacterial response. Therefore, we completed validation experiments using conventional or manual biochemical methods to assess key ions with regard to chromatogram shape, intensity and retention time, as well as collision-induced dissociation mass spectrometry (CID-MS) to confirm the identities of lipids initially established based on *m/z* values. Also, manual inspection of chromatograms shows all background signals, which helps to determine whether high fold-change events result from a complete or partial loss of unsaturated forms (Fig. 2B). For example, events corresponding to unsaturated mycobactins showed high fold-change in scatterplots (Fig. 2A, black diamonds), but it was unknown if all unsaturated mycobactins were lost after *mbtN* deletion. Manual generation of ion chromatograms for a representative unsaturated mycobactin (Fig. 2B, *m/z* 923.4701) showed baseline response in Δ*mbtN*, suggesting complete ablation.

Considering manual analysis of all targets, ion chromatograms corresponding to saturated (*m/z* 925.4858) and unsaturated (*m/z* 923.4701) mycobactin-iron holocomplexes ([M+Fe-2H]^+^), saturated (*m/z* 909.4909) and unsaturated (*m/z* 907.4754) monodeoxymycobactin-iron holocomplexes, saturated (*m/z* 840.5845) and unsaturated (*m/z* 838.5688) apoforms ([M+H]^+^) of dideoxymycobactins, and saturated (*m/z* 801.2878) and unsaturated (*m/z* 799.2722) carboxymycobactin-iron holocomplexes, corroborated the automated lipidomics results (Fig. 2B and S2 Fig). Specifically, all traces in wild type M. tb showed the expected sawtooth chromatogram shape, resulting from nearly co-eluting isobaric variants of mycobactins. Retention times were similar (±30 sec) to the known retention time for mycobactins of 5 to 6 min while carboxymycobactins eluted at 8 to 11 min (Fig. 2B and S2 Fig). Chromatograms from Δ*mbtN* of unsaturated mycobactin, monodeoxymycobactin, dideoxymycobactin and carboxymycobactin were at baseline, indicating a complete absence, while saturated forms were abundantly produced. Complementation of Δ*mbtN* restored production of unsaturated mycobactin and its deoxy forms (Fig. 2B).

CID-MS in comparison to authentic standards provides a basis for assigning each ion to a chemical structure and localizing the site of the altered saturation (Fig. 2C). Specifically, CID-MS detected characteristic fragments of unsaturated mycobactin (*m/z* 665.3506, *m/z* 355.0651) in wild type cultures and saturated mycobactin (*m/z* 667.3605, *m/z* 355.0634) in Δ*mbtN* cultures, consistent with the identity of compounds. Further, the collision patterns localize the site of altered mass to the fatty acyl unit and not to an unexpected portion of the polyketide-polypeptide unit (Fig. 2C), ruling in the action of the enzyme on the fatty acyl unit. These manual analyses further confirm certain aspects of the new lipidomics methods, and they prove that MbtN is the fatty acyl dehydrogenase that carries out an essential function in biosynthesis of unsaturated mycobactin and its deoxy forms.

### MbtK acts non-redundantly in siderophore biosynthesis

Next we carried out targeted and untargeted analyses of the 12,803 molecular events detected in Δ*mbtK* and its wild type parent. For all evaluable events, automated (Fig. 3A) and manual (Fig. 3B) analysis showed acylforms of mycobactins and their deoxy variants with signal intensity at or near zero in Δ*mbtK*. Signals were substantially restored with *mbtK* complementation. The corresponding substrate accumulation of mycobactin peptide was undetectable in lipid extracts; peptides are predicted to remain covalently attached to MbtE/F in the absence of MbtK [17,34,35]. Thus, despite the presence of many known or predicted acyl transferases in the M. tb genome, we conclude that MbtK acts non-redundantly in the transfer of saturated and unsaturated monocarboxylic and fatty acids to the lysine moieties of hydroxamate siderophores.

Turning to untargeted analysis of all detected molecular events, a strikingly large percentage, 74.2% of 12,803 measurements, met the criteria of two-fold signal intensity change (Fig. 3A). This degree of change is higher than that observed in the comparison of replicate, iron-depleted wild type cultures (S3 Fig) or Δ*mbtN* (Fig. 2A). Thus, MbtK deletion also causes an unexpectedly broad remodeling of M. tb’s lipid constituents, involving many types of lipids with masses and retention times that are unrelated to mycobactins.

### MbtK is necessary for growth during iron starvation and early virulence

**Fig 4:**
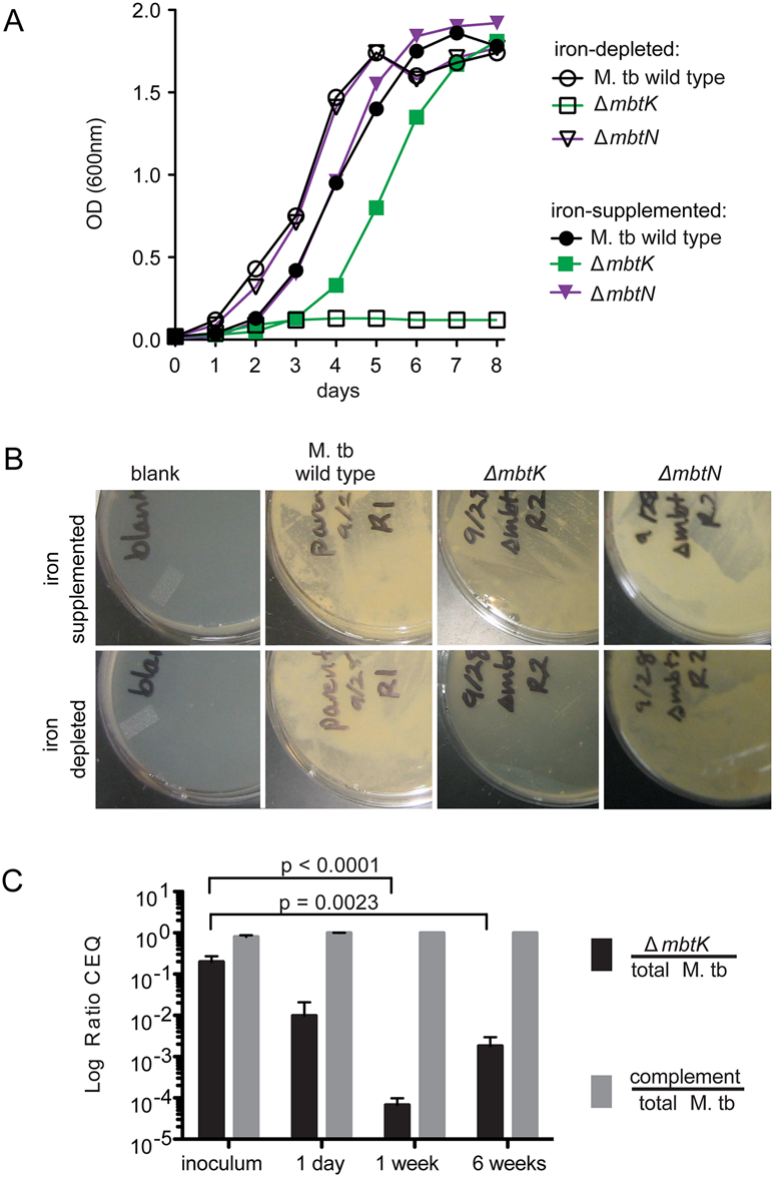
*mbtK* is required for growth during iron-starvation and early virulence *in vivo*. (**A**) Liquid M. tb cultures grown in iron-depleted medium, supplemented or not with 50 μM ferric chloride. (**B**) Representative plates from three replicates of the strains shown in (A) grown for 3 weeks on iron-depleted plates supplemented or not with 50 μM ferric chloride. (**C**) Δ*mbtK* and complemented Δ*mbtK*, marked chromosomally with unique identifiers (q-tags), were mixed 50:50 and used to infect fifteen C57/B6 mice at ~1,000 CFU via aerosol. Five mice were sacrificed at each time point. Lung homogenates were plated for CFU, colonies were counted and collected from plates to prepare genomic DNA. Average total recovered CFU were 1,131, 68,355 and 655,650 at 24 hours, 1 week and 6 weeks, respectively. Quantitative PCR for the q-tag specific to each strain was performed in duplicate, resulting in chromosomal equivalents (CEQ) of each strain to the total CEQ recovered per lung [37]. Log ratios were evaluated by unpaired T-tests.

Although MbtN is necessary for synthesis of unsaturated siderophores, the role, if any, of the unsaturation in iron scavenging and growth is unknown. For M. tb H37Rv Δ*mbtK*, we expected the absence of mycobactin to attenuate growth *in vitro* when heme iron was unavailable; however, production of biologically active unacylated mycobactin peptides or passive iron uptake might allow some iron scavenging. We grew Δ*mbtK* and Δ*mbtN* starter cultures in iron-depleted medium supplemented with 50 μM ferric chloride, washed them in iron-depleted medium, divided cultures in half, and inoculated each half into medium that was supplemented or not with iron. Whereas wild type and Δ*mbtN* grew well in iron-depleted medium, Δ*mbtK* was entirely unable to grow over eight days (Fig. 4A). Supplementation with 50 μM ferric chloride substantially rescued Δ*mbtK* growth, although a growth lag of two days was observed. Thus, Δ*mbtK* is essential for growth *in vitro* and the defect relates specifically to impaired iron scavenging. To confirm these results on solid medium, we plated the strains in triplicate on iron-depleted or iron-supplemented agar plates, and again observed scant growth of Δ*mbtK* on iron-depleted medium. On iron-supplemented agar medium, the relative growth defect of Δ*mbtK* was partially reversed (Fig. 4B). Thus, MbtK, but not MbtN, is required for non-heme iron acquisition and iron-dependent growth *in vitro*.

These *in vitro* assays provide iron in a non-heme form. However, *in vivo* iron can be derived from heme via the hemaphore pathway [4,5], or via scavenging of iron bound to host molecules. Understanding the relative roles of these mechanisms is important to guide development of pharmacological agents that might target these potentially overlapping pathways [6,36]. Therefore, mouse infection with Δ*mbtK* could provide insight into the necessity of mycobactins *in vivo*, and the relative importance of the mycobactin and hemophore pathways during infection. Fifteen C57/B6 mice were infected by aerosol with ~1,000 colony-forming units (CFU) of an equal mixture of Δ*mbtK* and complemented Δ*mbtK*. Prior to infection, each strain was chromosomally marked with a unique identifier (q-tag) to facilitate quantitative PCR (qPCR) assessment of survival *in vivo* [37]. Five mice were sacrificed at 24 hours, 1 week, and 6 weeks post-infection. Lung homogenates were plated and colonies collected for qPCR amplification specific to each q-tag, resulting in a log ratio of chromosomal equivalents (CEQ) for each strain compared to the total M. tb burden (Fig. 4C). Compared to the initial inoculum, there was a large (10 to 5,000-fold) decrease in Δ*mbtK* CEQs at all time points post-infection that met significance criteria at 1 (p< 0.0001) and 6 (p< 0.0023) weeks after infection. However, the pattern of response suggested that between 1 and 6 weeks post-infection, Δ*mbtK* CEQs began to recover. These results suggest that Δ*mbtK* has an early virulence attenuation for which other iron-acquisition systems are unable to compensate *in vivo*, but that MbtK is not absolutely required for growth *in vivo*.

### Unbiased analysis of MbtK effects during iron starvation

Next, we sought to understand the unexpectedly broad alterations in the lipid profile accompanying *mbtK* deletion (Fig. 3A). That 74 percent of all events would meet change criteria was surprising, because MbtK is thought to function only in the mycobactin/carboxymycobactin biosynthetic pathway [16,22]. Further, variations in culture conditions could not account for such global changes, as replicates of wild type M. tb cultures grown in parallel in iron-depleted medium generated a background change of 10.1% (S2 Fig). Our initial analysis of Δ*mbtK* showed that only a small fraction of the changed events corresponded to masses and retention times of mycobactin-like molecules (Fig. 3A). Therefore, we sought to take advantage of the full breadth of detection available in the lipidomic platform to investigate new mechanisms by which defective MbtK function could impact growth and virulence.

One explanation is that MbtK acts on substrates other than mycobactin peptides to generate currently unknown lipopeptides with functions unrelated to the iron scavenging effects of mycobactins. If true, these substrates would accumulate and products disappear in Δ*mbtK*. Such changes in the intensity of these events would be expected regardless of the presence of iron, and they would be rescued by *mbtK* complementation, similar to the pattern observed with mycobactin (Fig. 3B). A second general type of ion that could accumulate is unacylated mycobactin peptide. To determine if substrate accumulation could account for these types of lipid changes, we calculated the *m/z* of mycobactin peptide substrates of MbtK and determined their approximate retention time. Molecular events with masses and retention times corresponding to unknown upstream substrates of MbtK were absent from both Δ*mbtK* and wild type cells, suggesting substrate accumulation was not the sole cause of intensity changes. The absence of mycobactin peptides in lipid extracts was expected, as they are predicted to remain covalently bound to MbtE or MbtF, the enzymes preceding MbtK in the mycobactin biosynthetic pathway [17,34,35]. Further, intensities of many molecular events decreased in Δ*mbtK* could be rescued simply by supplementing cultures with iron. Thus, we could not rule in that altered ions in Δ*mbtK* were unknown products/substrates of MbtK, but more likely are downstream products appearing due to iron starvation.

To our knowledge, iron depletion is not directly linked to altered lipid metabolism in M. tb. However, iron is necessary for oxidative phosphorylation and other core metabolic pathways that might broadly influence downstream events in lipid metabolism. Therefore, we next considered the hypothesis that extreme iron deficiency, due to the absence of mycobactin in Δ*mbtK*, combined with growth in iron-depleted medium, creates stress that alters lipids that are structurally unrelated to mycobactins, but whose abundance is regulation by mycobactin and iron. We did not have the experimental resources to investigate 12,803 targets (Fig. 3A), so we used bioinformatic strategies to prioritize targets. The molecular events we focused on correspond to known molecules with high fold-changes of intensity between iron-starved Δ*mbtK* and iron-starved wild type bacteria. To generate this list of targets, we first excluded events corresponding to unknown lipids, focusing on molecular events matching known families of lipids in the MycoMap database [28].

We next extended the two-way analysis that compared wild type and Δ*mbtK* in iron-depleted medium (Fig. 3A), to a four-way analysis that also considered iron supplementation and genetic complementation. This analysis sought to identify those events that were altered by *mbtK* deletion, but rescued by chemical complementation with iron and by genetic *mbtK* complementation.

Next, we used mass interval criteria to group molecular events corresponding to a lipid family. This approach is based on the fact that most lipid families (i.e., polyketides, mycolic acids, fatty acids) occur as chain length variants, which differ by an integer number of CH_2_ units. Therefore, ions from one lipid family can be readily recognized as alkane series members when they differ by an integer number of *m/z* 14.0156. As illustrated in a study of global lipid changes occurring in response to hypoxia [38], biological regulation by an upstream stimulus can be distinguished from random variation in intensity signals because most or all members of a family are typically regulated in parallel.

Finally, rather than compiling all events meeting a minimal 2-fold change in intensity, we ranked molecular events and analyzed those with the highest fold-changes, often exceeding 100-fold. Thus, the focusing strategy applied to 12,083 events sought to identify the particular subset of events corresponding to families of lipids that are strongly regulated by MbtK expression and iron availability.

### MbtK deletion depletes membrane phospholipids during iron starvation

**Fig 5:**
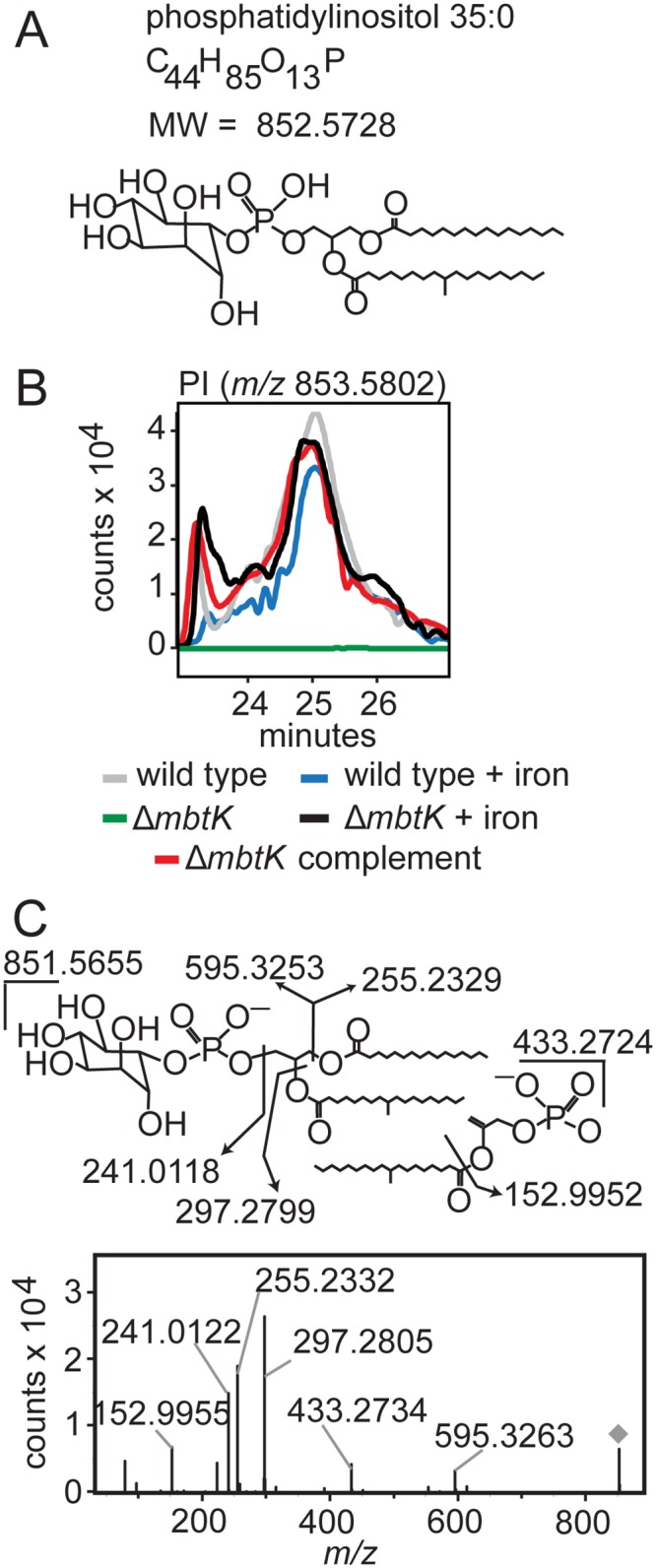
*mbtK* deletion depletes phosphatidylinositol during iron starvation. **(A)** Phosphatidylinositol C35:0, neutral mass 852.5728, was detected in the positive mode (**B**) as [M+H]^+^ (*m/z* 853.5802) from triplicate total lipid extracts that were normalized for mass. Chromatogram is representative of triplicate runs. (**C**) Collision of phosphatidylinositol C35:0 in the negative mode as the [M-H]^-^ ion, *m/z* 851.5655, showed the expected fragments confirming its structure.

This organism-wide lipidomic search returned a remarkably consistent pattern of significantly decreased signals corresponding to cytoplasmic membrane phospholipids in Δ*mbtK*, as compared to similarly grown wild type or genetically or chemically complemented mutants. Analysis of molecular feature, *m/z* 853.5802, illustrates the discovery process. This event matches the known mass of the phosphatidylinositol (PI) ion [M+H]^+^, C_44_H_85_O_13_P, containing 35 carbon atoms and no unsaturations (C35:0) in its two alkyl chains (Fig. 5A). This event has average intensity counts of 2.1 x 10^6^ in iron-starved wild type, 1.9 x 10^6^ in iron-supplemented wild type, 0.3 x 10^6^ in iron-starved Δ*mbtK*, 2.2 x 10^6^ in iron-supplemented Δ*mbtK*, and 2.3 x 10^6^ in iron-starved Δ*mbtK* complement. Therefore, it meets the criteria for high fold-change and intensity rescue by iron supplementation and complementation. The changes in intensity of PI C35:0 across the five conditions were compared to events corresponding to other PI acyl forms and there were no nearly co-eluting molecules that matched alternatively acylated PI forms. The intensities of these events showed similar patterns among the five conditions that track in parallel with one another and with PI C35:0. The pattern of relative abundance of PI acylforms in wild type and Δ*mbtK* was similar, suggesting that absence of MbtK did not simply induce a shift in abundance from one acylform to another (S1 Table). Further, key aspects of this computerized analysis were validated in manual analysis of ion chromatograms, which were consistent with a single molecule at the expected retention time for phosphatidylinositol C35:0 (Fig. 5B). CID-MS of *m/z* 851.5655 was consistent with the [M-H]^-^ ion of phosphatidylinositol C35:0 (Fig. 5C). Although phospholipid concentration is not measured directly in this high throughput method, the intensity count values for time-of-flight MS detection reliably correlate with mass input over a broad concentration range of mycobacterial lipids [28]. Collectively, these data show that the approximately ten-fold change in PI intensity results from *mbtK* deletion in the setting of iron starvation.

**Fig 6:**
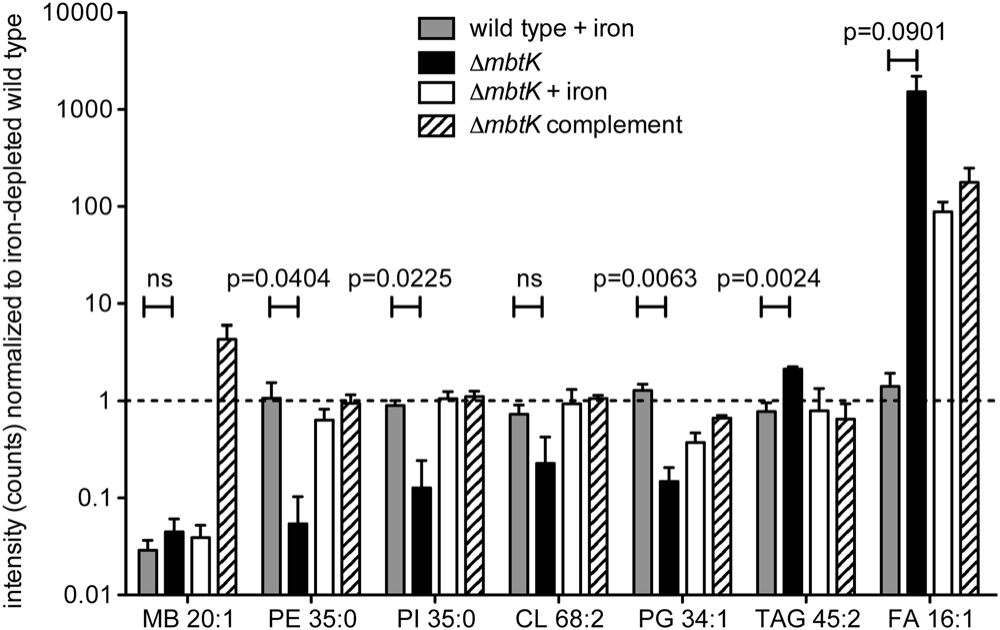
*mbtK* deletion decreases phospholipid abundance during iron starvation. Mean intensities of representative lipids from 5 glycerophospholipid classes and mycobactin control were normalized to their value in triplicate iron-depleted wild type cultures grown and analyzed in triplicate. Dashed line indicates the ion intensity in iron-depleted wild type. Statistical comparisons are Student’s t-tests of ion intensities in iron-supplemented wild type and iron-depleted Δ*mbtK*. MB = mycobactin, PE = phosphotidylethanolamine, PI = phosphotidylinositol, CL = cardiolipin, PG = phosphatidylglycerol, TAG = triacylglyceride, FA = fatty acid.

By repeating this process across the five conditions, and for events corresponding to known molecules, we observed highly similar patterns of MbtK regulation of every major phospholipid class in the cytoplasmic membrane of M. tb, including phosphatidylethanolamine (PE), cardiolipin (CL), phosphatidylglycerol (PG), and triacylglyceride (TAG) (Fig. 6 and S4 Fig). As measured by average ion intensity, the degree of change is high in iron-starved Δ*mbtK*, with loss of 50 to 90 percent for phospholipids. Finally, in all cases lipid intensity is rescued by *mbtK* complementation or iron supplementation.

This analytic method measures steady state phospholipid pools and does not distinguish between reduced phospholipid synthesis or increased catabolism. However, phospholipid catabolism in response to hypoxic, redox and other stresses has been described previously and is known to occur through the action of phospholipases, which act on abundant membrane phospholipids, generating free fatty acid [39,40]. Consistent with this scenario, we observed signal increases for triacylglyceride and free fatty acids in iron-starved Δ*mbtK*, which were rescued with iron supplementation or complementation (Fig. 6).

To determine whether redox stress occurred in the Δ*mbtK* mutant, we scanned lipidomics datasets for ions annotated as menaquinone-9 (C_56_H_80_O_2_, neutral mass 784.6158), a key component of the M. tb electron transport chain (S4 Fig). Menaquinone is the primary quinone in mycobacteria, converting between oxidized menaquinone and reduced menaquinone-H_2_ [41,42]. Annotation of the existing lipidomic dataset identified ions matching the calculated mass of the [M+Na]^+^ adducts of oxidized and reduced menaquinone-9 in lipid extracts at their expected retention time range of 4–8 mins [28]. Wild type M. tb grown in iron-depleted medium generated slightly more of the putative oxidized (*m/z* 807.6050) than reduced menaquinone-H_2_ (*m/z* 809.6207); however, reduced menaquinone-H_2_ was substantially more abundant than oxidized menaquinone in iron-depleted Δ*mbtK*. Complementation of Δ*mbtK* rescued this phenotype, suggesting *mbtK* deletion causes an imbalance of putative oxidized and reduced menaquinone. These results show how *post facto* analysis of organism-wide datasets can be interrogated to understand the downstream events controlling lipid metabolism, pointing toward an unexpected but massive iron-induced depletion of membrane phospholipids and an increased pool of free fatty acids.

## Discussion

When the substrates, cofactors and products of enzymes can be predicted, the reactions they catalyze can be reliably and quantitatively assessed *in vitro*. Such classical enzymology approaches determine the reactions for which an enzyme is sufficient. These methods predicted that MbtN and MbtK function as a mycobactin dehydrogenase and acyl transferase, respectively [16,22]. By combining gene deletion with new profiling platforms, it is possible to determine the diverse biochemical reactions for which an enzyme is necessary in its biological context, based on the natural substrates to which it is exposed [15,26,28]. Further, this approach can measure the relative contribution of intact pathways that operate in parallel, providing useful benefits to drug discovery, which relies on identification of non-redundant pathways. For example, M. tb expresses over 250 genes predicted to function in lipid metabolism [43]. In many cases, acyl transferases have highly overlapping predicted functions, making it difficult to identify the acyl transferase required for production of one particular lipid [44,45]. However, the strategy of lipidomic profiling shows that MbtK is absolutely required for its biochemical role in mycobactin biosynthesis as well its biological role in iron capture *in vitro* and *in vivo*.

Given the essentiality of iron to infection, it is logical that multiple iron acquisition pathways would be active during M. tb infection. The discovery of the hemophore system [4,5] raises questions about its redundancy *in vivo* with the mycobactin-carboxymycobactin pathway, and the role each plays in virulence. A mutant early in the mycobactin biosynthesis pathway (*mbtE*) is unable to grow *in vivo* [10]; accordingly, our infection study indicates strong growth attenuation of Δ*mbtK* in mice at early time points, with some restoration of growth later in infection. Growth attenuation is not as extreme as Δ*mmpS4/5*, which is expected because that mutant lacks the siderophore export apparatus and accumulates toxic siderophore intermediates, while Δ*mbtK* does not [46]. Our data suggest that mycobactin supports growth during M. tb’s paucibacillary stage, but that its necessity during later time points of *in vivo* growth lessens. The emergence of adaptive immunity after two weeks could affect Δ*mbtK* growth. A second, more favored scenario to explain the differing roles of MbtK in these settings is that iron is absolutely required for growth in general, and that mycobactin and heme transporters are both somewhat important *in vivo*, but have distinct roles during the early and late stages of infection, respectively. Other known aspects of the pathophysiology of tuberculosis are consistent with this hypothesis. During early stages of paucibacillary growth, M. tb would be expected to acquire iron from stores in undamaged tissue and invading macrophages. Later stages of tuberculosis cause hemorrhagic lung disease, which could potentially provide heme-bound iron [5]. Increasing evidence supports the hypothesis that paucibacillary growth at the earliest stages of infection can be decisive for transmission and medical intervention [47–50], so inhibitors of mycobactin function might be considered as therapeutic agents in this setting.

These data show that MbtN plays an essential biochemical role in creating unsaturated mycobactins and carboxymycobactins. However, the unsaturation is not required for normal growth *in vitro*. Prior studies have determined the unusual location and nature of the *cis* C2-3 unsaturation, present in the carboxyl unit of mycobactin and dicarboxyl unit of carboxymycobactin, which is thought to be unique among acylated lipids in M. tb [23,24]. Even if unnecessary for iron uptake *in vitro*, evolutionary conservation of this unusual unsaturation suggests that it has some biological function. This unsaturation might affect the cellular handling of mycobactin-like molecules, or the known effect of the unsaturation in increasing immunogenicity of dideoxymycobactin, a CD1a-presented T cell antigen, may be a natural but non-nutritional function [25].

Another unexpected conclusion derived from the lipidomics datasets is the broad and marked reduction in pools of membrane phospholipids in iron-starved Δ*mbtK*. These changes were accompanied by increased signals for free fatty acids, triacylglyceride and menaquinone-H_2_, suggesting the bacteria are metabolically responding to redox stress. To our knowledge, direct connections of iron depletion to mycobacterial phospholipid biosynthesis are not documented, but our findings are consistent with published reports that iron-starved M. tb induce a cascade of genes involved in stress responses, which may secondarily affect phospholipid pools [18,51]. However, the pool size of phospholipids and fatty acids changed in opposite ways, which might be explained by release of fatty acids in response to stress [27,51].

Rescue of the Δ*mbtK* lipid phenotype by iron suggests a connection between iron starvation stress and lipid metabolism, but does not describe a specific mechanism. Consistent with the increased triacylglyceride signal in iron-starved Δ*mbtK*, previous studies have demonstrated M. tb accumulation of triacylglyceride during redox stress via DosR or WhiB3 signal transduction [12,52,53]. Further, redox stress from vitamin C treatment decreased phospholipids in M. tb [40]. Iron can play several roles in maintaining redox balance. For example, iron is an essential component of iron-sulfur clusters in electron transport chain cytochromes and dehydrogenases, which regenerate reducing equivalents such as NAD^+^/NADH [54]. The inability to oxidize reducing equivalents may cause “reductive stress,” leading to build up of electrons in the menaquinone pool and excess menaquinone-H_2_. Further, the extreme iron starvation that occurs in the absence of MbtK may cause phospholipid catabolism to continue unabated, resulting in decreased plasma membrane integrity and death of some cells. However, the low phospholipid state is not merely a death phenotype, as live bacteria can be grown from affected cultures and infected mice. Our results are consistent with upregulated phospholipid catabolism and glycerol lipid accumulation to ensure a ready supply of carbon.

Our data place MbtK at the crossroads of three M. tb phenotypes required for pathogenesis: the ability to acquire iron *in vivo*, maintenance of phospholipids, and growth in the lung. Affecting all three phenotypes by removing a single enzyme supports targeting of MbtK or other steps in mycobactin synthesis as a therapy for tuberculosis [55]. Further, the unexpected lipid response of M. tb to *mbtK* deletion was extrapolated using lipidomics as a new approach to describe enzyme function in biological context. Thus, lipidomic profiling provides broad and unexpected insight into mechanisms by which the primary enzymatic mechanism of MbtK—lipid transfer to a peptide—controls a cascade of downstream effects involving mycobactin synthesis, iron depletion and lipid catabolism.

## Materials and Methods

### Bacterial strains and growth conditions

To construct M. tb H37Rv deletion mutants in *mbtK* or *mbtN*, 1 kilobase regions of the genome flanking each gene were amplified and cloned into pJM1, a counterselectable mycobacterial suicide vector containing genes for chloramphenicol resistance, hygromycin resistance (*hyg*), and sucrose sensitivity (*sacB*). Transformants having undergone single crossover events were selected on hygromycin. PCR with primers within *hyg* (5′-GAATCCCTGTTACTTCTCGACCGT-3′ and 5′-AGGTCCACG AAGATGTTGGTCC-3′) confirmed single crossover events. Transformants were plated on mycobactin-containing sucrose medium to select for double crossover events; PCR with internal primers (*mbtK*: 5′-TCATGCTCACCGAGGAACTTGCA T-3′ and 5′-AGA TGTTGGCGGAGTGGATGAA-3′; *mbtN*: 5′-TCGGTAAACTCGTCGAACTCGCTT-3′ and 5′-CGAAGATGTGCATGCATTCGGAGA-3′) and external primers (*mbtK*: 5′-ATGATGAGTCGACGTCAGTTCGGT-3′ and 5′-AGGGACTCGAACCCTCAAAC TCTT-3′; *mbtN*: 5′-ATGCAAGTTCCTCGGTGAGCATGA-3′ and 5′-AGCCGTGAA ATTGGCGAAATCGAG-3′) confirmed deletions. To complement, *mbtK* or *mbtN* were expressed under the mycobacterial *groEL* promoter on integrating plasmid pGH1000A [31] and confirmed by PCR. Restoration of mycobactin production was confirmed by HPLC-MS.

For comparative lipidomics, three 5 ml cultures of M. tb H37Rv parent and mutant strains were grown to log phase in a defined iron-depleted medium containing 0.5% (w/v) KH_2_PO_4_, 2% (v/v) glycerol, 0.5% (w/v) L-asparagine and 10% albumin-dextrose-sodium chloride complex (ADN) [18,56]. Medium containing 0.5% KH_2_PO_4_, 2% glycerol and 0.5% L-asparagine was incubated overnight with 5% (w/v) Chelex-100 (Bio Rad) to lower the trace metal concentration. After removing Chelex by filtration, the pH was adjusted to 6.8 and medium was supplemented with 10% ADN, 0.5 mg ZnCl_2_, 0.1 mg MnSO_4_, and 40 mg MgSO_4_ per liter. For assaying growth on plates, 50 μl from each initial culture was spread onto an iron-depleted or iron-supplemented plate, containing 1.5 g agar (Bacto) and 100 mg cyclohexamide per liter. Iron-supplemented plates contained 50 μM FeCl_3_.

### Lipidomics

To extract cell lipids, bacteria from triplicate plates were treated with 60 ml 2:1 (V:V), then 1:1, then 1:2 chloroform:methanol for 1 h each. Extractable lipids were separated by centrifugation, collected and dried. Acetone insoluble cell lipids were made by collecting precipitates from 30 mg total lipids per 0.21 ml 4°C acetone on ice for 1 h, followed by washing with 4°C acetone. Acetone precipitates were washed 3 times with 4°C acetone; supernatants and washes were collected and dried to yield acetone soluble cell lipids, which were mass-normalized and analyzed in triplicate by a reversed phase method [15] on an Agilent 6520 Accurate Mass QToF mass spectrometer. Acetone insoluble lipids were analyzed by normal phase HPLC-MS as previously described [28].

Supernatant lipids from triplicate liquid M. tb cultures, used to measure carboxymycobactin production, were extracted with an equal volume of ethyl actetate, dried, mass-normalized, and analyzed by reversed phase HPLC-MS as previously described [15].

### Mouse infection

Fifteen mice received ~1,000 colony-forming units (CFU) of an estimated 50:50 mixture of q-tagged Δ*mbtK* and complemented Δ*mbtK* via aerosol. Five mice were sacrificed at 1 day, 1 week, and 6 weeks post-infection. Lung homogenates were plated for CFU, colonies were counted and then scraped from plates to prepare genomic DNA. Quantitative PCR was performed in duplicate with individual primers and probes designed to recognize each strain’s q-tag [37]. Log ratios were evaluated by unpaired T-tests.

### Ethics statement

This study was performed in strict accordance with the recommendations in the Guide for the Care and Use of Laboratory Animals of the National Institutes of Health. The protocol was approved on June 12^th^, 2013 by the Harvard Medical Area Standing Committee on Animal Care and Use (Accreditation #000009).

## Supporting Information

S1 Fig Genomic sequence of mycobactin synthase mutants. Sequencing results from Δ*mbtN* **(B)** or Δ*mbtK* **(C)** compared to the wild type locus **(A)** show in-frame, null deletions of *mbtN* or *mbtK*.
(TIF)

S2 Fig Carboxymycobactin production by Δ*mbtN* and Δ*mbtK*. Deletion of *mbtK* or *mbtN* abrogates carboxymycobactin production. (**A**) Chromatograms of carboxymycobactin C9:1, detected as [M-2H+Fe]^+^, in the unsaturated (*m/z* 799.2722) or saturated (*m/z* 801.2878) form. Chromatograms are representative of triplicate iron-depleted cultures conditioned for two weeks to stimulate carboxymycobactin production. (**B**) Representative ion chromatograms (left) from three experiments and collision-induced dissociation (right) of unsaturated carboxymycobactin (*m/z* 785.2558) from wild type, and saturated carboxymycobactin (*m/z* 787.2951) from Δ*mbtN*. Structures are labeled with calculated masses, while mass spectra show detected ions. The constant presence of *m/z* 355.0614, corresponding to the polyketide-polypeptide backbone, is accompanied by a characteristic two-mass unit difference in unsaturated and saturated fragments separating the phenoloxazoline and acyl chain from the cobactin (*m/z* 527 and 529), isolating the mass difference to the fatty acyl unit. Collided molecules indicated by grey diamonds. (**C**) Chromatograms of carboxymycobactin C9:1 from iron-depleted Δ*mbtK* cultures.
(TIF)

S3 Fig Culture-to-culture variation in lipid detection. Lipidomic comparison of two wild type cultures grown in iron-depleted medium, analyzed in triplicate and represented as in Fig. 2A, show culture-to-culture intensity variation for mycobactins and their deoxy forms. Highlighted ions match the highlighted calculated *m/z* values in Fig. 1C, corresponding to: saturated mycobactins (white diamonds; *m/z* 911.4739, *m/z* 925.4883 and *m/z* 939.5008), unsaturated mycobactins (black diamonds; *m/z* 909.4564, *m/z* 923.4726 and *m/z* 937.4869), saturated monodeoxymycobactins (white circles; *m/z* 842.5663, *m/z* 856.5809, *m/z* 856.5828 and *m/z* 881.4590), unsaturated monodeoxymycobactins (black circles; *m/z* 865.4293, *m/z* 893.4444, and *m/z* 907.4781), saturated dideoxymycobactins (white squares; *m/z* 826.5531 and *m/z* 840.5852), and unsaturated dideoxymycobactins (black squares; *m/z m/z* 824.5594 and *m/z* 838.5714). Ions between the dashed lines have less than a two fold-change in intensity between replicates.
(TIF)

S4 Fig Changes in lipid abundance in Δ*mbtK*. (**A**) Representative ion chromatograms from triplicate HPLC runs of the acetone insoluble fraction from triplicate cultures of wild type (grey) or Δ*mbtK* (green) grown in iron-depleted medium, detected by normal phase HPLC-MS. Features were aligned and analyzed as in Fig. 2, with MycoMass used to identify cardiolipin, phosphatidylglycerol, fatty acid, triacylglyceride, and phosphatidylethanolamine. (**B**) Average abundance of oxidized (white bars) or reduced (black bars) menaquinone-9 in triplicate iron-depleted cultures of wild type, Δ*mbtK*, or Δ*mbtK* complement.
(TIF)

S1 Table Phosphatidyl inositol abundances. Intensities (counts) of unsaturated phosphatidylinositol acylforms from three replicate cultures of wild type and Δ*mbtK* grown in iron-depleted medium.
(XLSX)

S2 Table Lipid abundances. (**A**) Log-transformed intensities from Fig. 6 (wild type, Δ*mbtK* or Δ*mbtK* complement grown in iron-depleted medium; Δ*mbtK* grown in iron-supplemented medium). (**B**) Comparison of ion intensities (Student’s t-test) in Δ*mbtK* grown in iron-supplemented medium to Δ*mbtK* grown in iron-depleted medium (left), or Δ*mbtK* grown in iron-depleted medium to wild type grown in iron-depleted medium (right).
(XLSX)
