# Supplementary figures and images for: Lipidomic Analysis Links Mycobactin Synthase K to Iron Uptake and Virulence in M. tuberculosis
Source: PLoS Pathog. 2015 Mar 27;11(3):e1004792. doi: 10.1371/journal.ppat.1004792 (PMC4376628; doi:10.1371/journal.ppat.1004792)

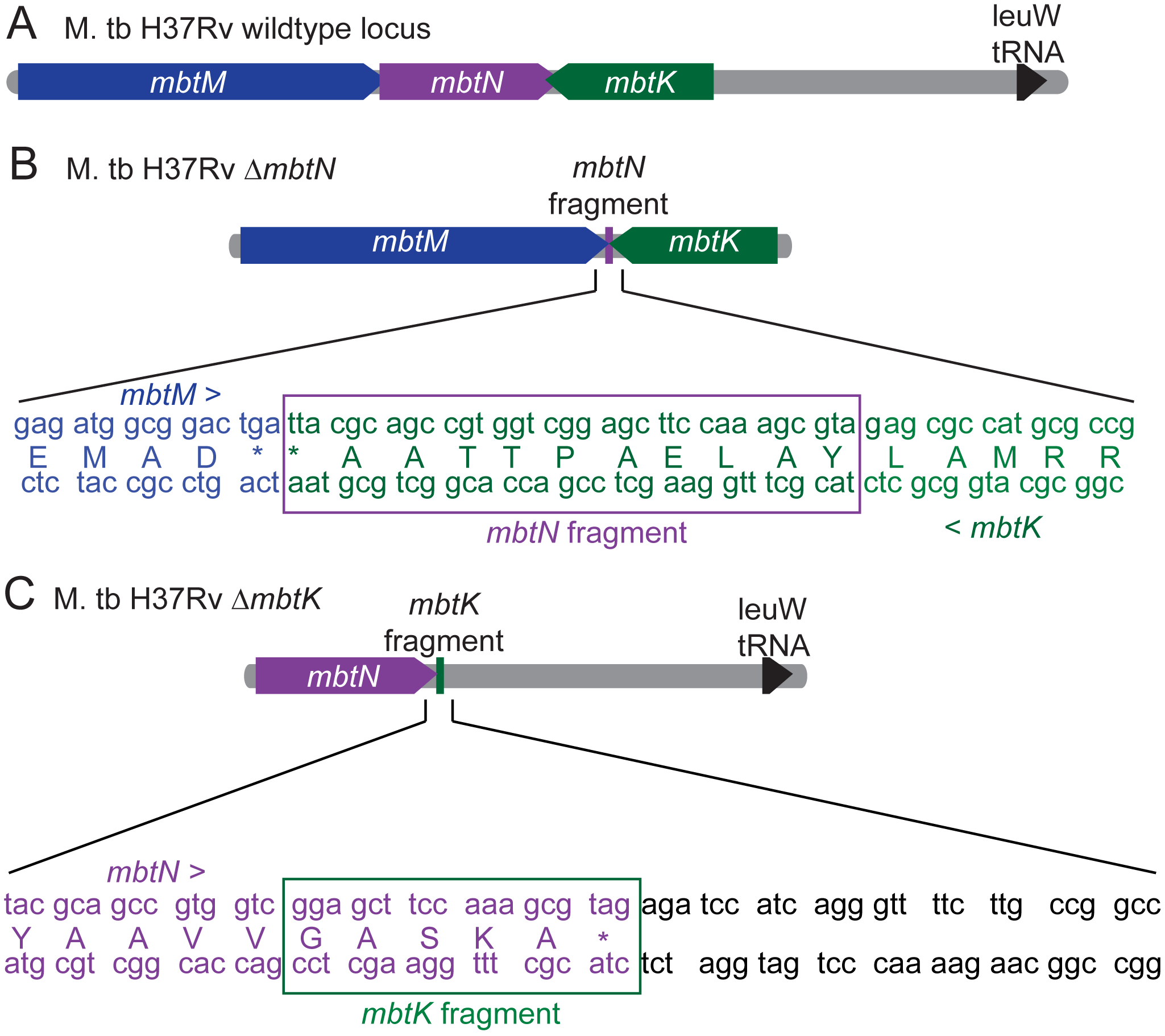

Supplement: S1 Fig — Sequencing results from ΔmbtN (B) or ΔmbtK (C) compared to the wild type locus (A) show in-frame, null deletions of mbtN or mbtK. (TIF) [file ppat.1004792.s001.tif]

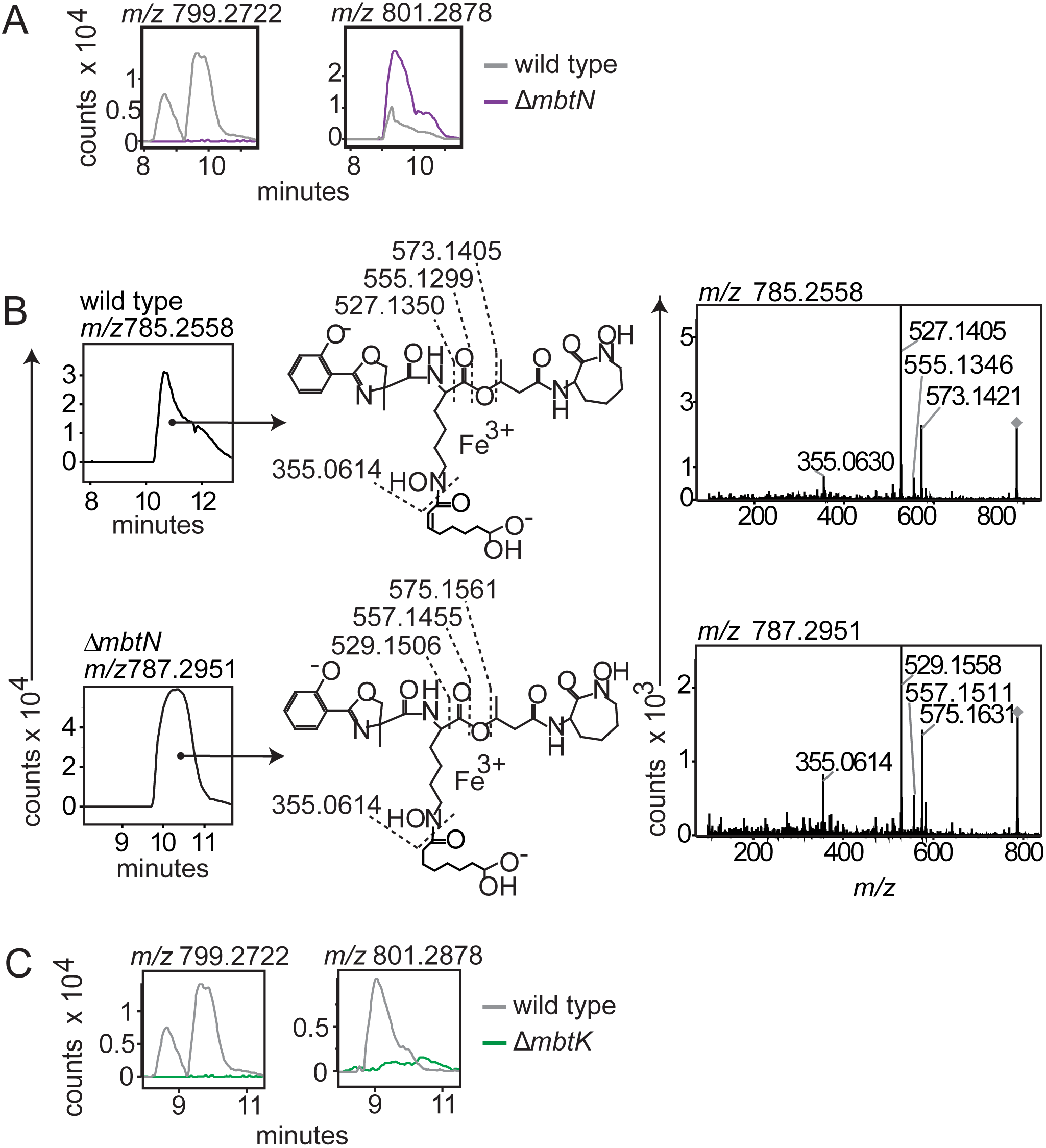

Supplement: S2 Fig — Deletion of mbtK or mbtN abrogates carboxymycobactin production. (A) Chromatograms of carboxymycobactin C9:1, detected as [M-2H+Fe]+, in the unsaturated (m/z 799.2722) or saturated (m/z 801.2878) form. Chromatograms are representative of triplicate iron-depleted cultures conditioned for two weeks to stimulate carboxymycobactin production. (B) Representative ion chromatograms (left) from three experiments and collision-induced dissociation (right) of unsaturated carboxymycobactin (m/z 785.2558) from wild type, and saturated carboxymycobactin (m/z 787.2951) from ΔmbtN. Structures are labeled with calculated masses, while mass spectra show detected ions. The constant presence of m/z 355.0614, corresponding to the polyketide-polypeptide backbone, is accompanied by a characteristic two-mass unit difference in unsaturated and saturated fragments separating the phenoloxazoline and acyl chain from the cobactin (m/z 527 and 529), isolating the mass difference to the fatty acyl unit. Collided molecules indicated by grey diamonds. (C) Chromatograms of carboxymycobactin C9:1 from iron-depleted ΔmbtK cultures. (TIF) [file ppat.1004792.s002.tif]

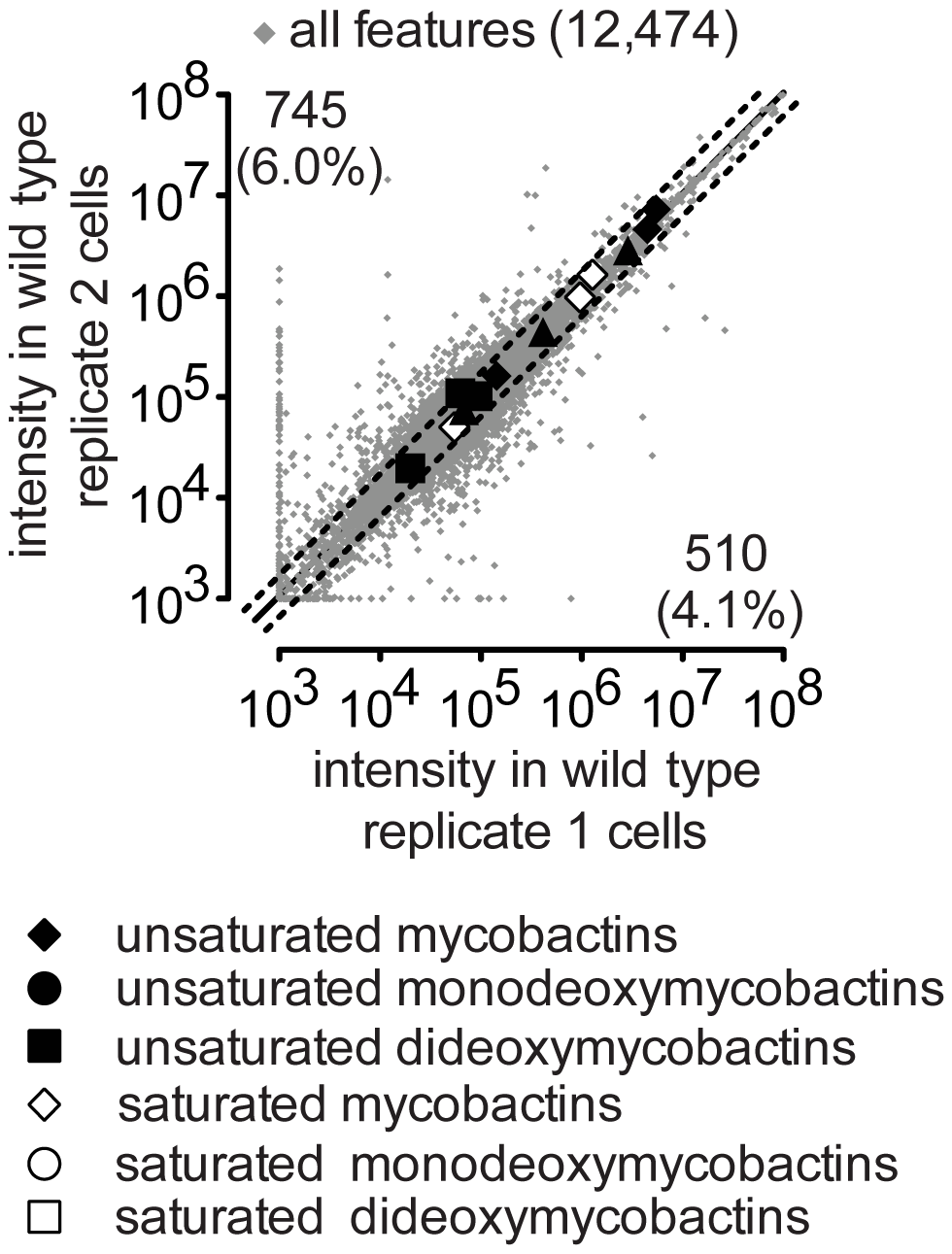

Supplement: S3 Fig — Lipidomic comparison of two wild type cultures grown in iron-depleted medium, analyzed in triplicate and represented as in Fig. 2A, show culture-to-culture intensity variation for mycobactins and their deoxy forms. Highlighted ions match the highlighted calculated m/z values in Fig. 1C, corresponding to: saturated mycobactins (white diamonds; m/z 911.4739, m/z 925.4883 and m/z 939.5008), unsaturated mycobactins (black diamonds; m/z 909.4564, m/z 923.4726 and m/z 937.4869), saturated monodeoxymycobactins (white circles; m/z 842.5663, m/z 856.5809, m/z 856.5828 and m/z 881.4590), unsaturated monodeoxymycobactins (black circles; m/z 865.4293, m/z 893.4444, and m/z 907.4781), saturated dideoxymycobactins (white squares; m/z 826.5531 and m/z 840.5852), and unsaturated dideoxymycobactins (black squares; m/z m/z 824.5594 and m/z 838.5714). Ions between the dashed lines have less than a two fold-change in intensity between replicates. (TIF) [file ppat.1004792.s003.tif]

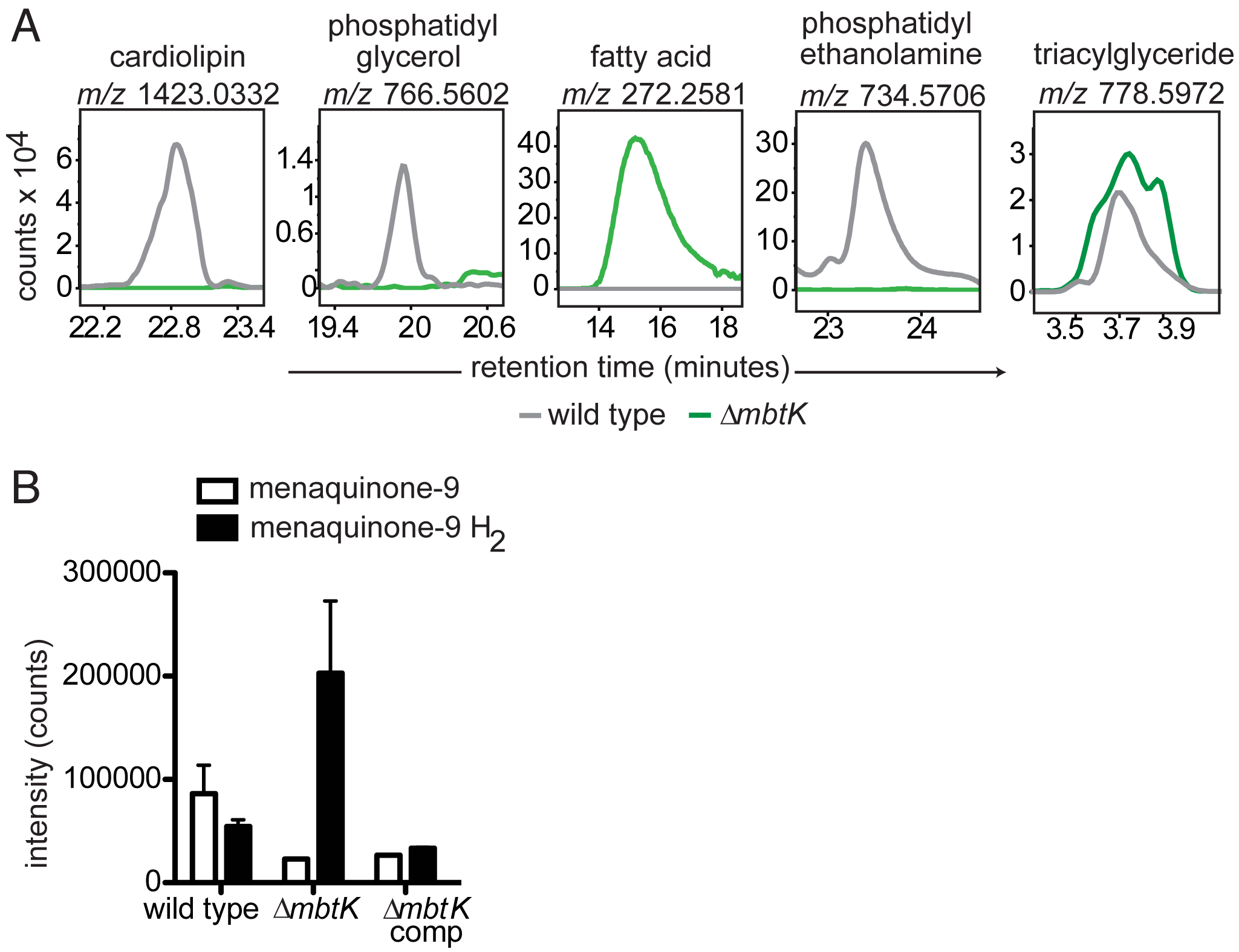

Supplement: S4 Fig — (A) Representative ion chromatograms from triplicate HPLC runs of the acetone insoluble fraction from triplicate cultures of wild type (grey) or ΔmbtK (green) grown in iron-depleted medium, detected by normal phase HPLC-MS. Features were aligned and analyzed as in Fig. 2, with MycoMass used to identify cardiolipin, phosphatidylglycerol, fatty acid, triacylglyceride, and phosphatidylethanolamine. (B) Average abundance of oxidized (white bars) or reduced (black bars) menaquinone-9 in triplicate iron-depleted cultures of wild type, ΔmbtK, or ΔmbtK complement. (TIF) [file ppat.1004792.s004.tif]
